# Seroprevalence and Risk Factors for Hepatitis E Virus in a Metropolis of Northeastern Brazil: A Population‐Based Survey

**DOI:** 10.1111/zph.70050

**Published:** 2026-03-09

**Authors:** Carolline A. Mariz, Cynthia Braga, Wayner V. Souza, Carlos F. Luna, André Luiz Sá de Oliveira, Elisa de Almeida Neves de Azevedo, Clarice N. L. de Morais, Maria de Fatima P. M. Albuquerque, Edmundo Pessoa Lopes

**Affiliations:** ^1^ Department of Parasitology, Institute Aggeu Magalhães Oswaldo Cruz Foundation Recife Pernambuco Brazil; ^2^ Department of Public Health, Institute Aggeu Magalhães Oswaldo Cruz Foundation Recife Pernambuco Brazil; ^3^ Department of Geoprocessing, Institute Aggeu Magalhães Oswaldo Cruz Foundation Recife Pernambuco Brazil; ^4^ Department of Virology, Institute Aggeu Magalhães Oswaldo Cruz Foundation Recife Pernambuco Brazil; ^5^ Postgraduate Program in Tropical Medicine, Center of Medical Sciences Universidade Federal de Pernambuco (UFPE) Recife Pernambuco Brazil; ^6^ Division of Gastroenterology, Hospital das Clínicas/EBSERH Universidade Federal de Pernambuco (UFPE) Recife Pernambuco Brazil

**Keywords:** anti‐HEV, epidemiology, hepatitis E, prevalence, public health

## Abstract

**Introduction:**

Hepatitis E virus (HEV) is a leading cause of acute hepatitis worldwide. While traditionally linked to poor sanitation in endemic areas, evidence shows increasing circulation in developed settings. In Brazil, however, population‐based data remain limited. Therefore, this study aimed to estimate HEV seroprevalence and identify associated risk factors in the general population of a metropolis in Northeastern Brazil.

**Methods:**

Serum samples from a population‐based survey using stratified cluster sampling and multistage selection were tested for anti‐HEV (IgG) by ELISA. IgG‐positive cases were further tested for IgM. Risk factors were analysed by odds ratio (OR) through bivariate logistic regression, considering *p* < 0.05 significant. Estimates were weighted by sample design effect using the ‘survey’ package (v4.1–1). Analyses were performed in Stata 15.

**Results:**

Among 2,070 samples, 74 were positive for anti‐HEV IgG (3.6%; 95% CI: 2.9–4.3), with no IgM reactivity. Seroprevalence by socioeconomic stratum was 3.3% (95% CI: 1.7–5.0) in the high stratum, 3.8% (95% CI: 2.6–4.6) in the intermediate and 3.5% (95% CI: 2.4–4.6) in the deprived stratum. Adults aged 55–65 years had higher odds of previous HEV exposure (adjusted OR = 6.02; 95% CI: 2.59–13.99; *p* < 0.001) compared to the 5–24 year group. Residents in households with alternative sewage systems showed higher odds of HEV positivity than those with public sewage access (OR = 1.58; 95% CI: 1.08–2.31; *p* = 0.021).

**Conclusion:**

Our findings demonstrate HEV circulation in metropolitan Northeast Brazil. Exposure was primarily associated with older age and precarious sanitary infrastructure, highlighting the need for targeted public health surveillance and improved basic sanitation.

## Impacts

- Traditionally associated with outbreaks in endemic regions characterised by inadequate sanitation, hepatitis E virus (HEV) infection is increasingly recognised as circulating in developed countries. In Brazil, however, population‐based data on HEV remain scarce.
- To date, no information has been available regarding HEV seroprevalence and related risk factors in the general population of a major metropolitan area in Northeastern Brazil.
- This study, conducted for the first time in Recife, identifies the seroprevalence of HEV among the general population. Older individuals and those residing in areas lacking public sewage infrastructure were at higher risk of infection, underscoring the need for targeted preventive strategies and improved sanitation measures.

## 1 Introduction

Hepatitis E virus (HEV) is a leading cause of acute viral hepatitis worldwide, with an estimated 900 million people having been exposed and 15–110 million experiencing recent or ongoing infections annually (Li et al. 2020). The clinical spectrum of HEV infection is broad, ranging from asymptomatic cases (approximately 70%) to self‐limiting acute hepatitis. However, it can progress to severe outcomes including fulminant hepatitis, particularly in pregnant women and chronic infection in immunocompromised individuals (Lhomme et al. 2020; Velavan et al. 2021). To date, HEV‐induced acute liver failure (ALF) has not been documented among pregnant women in Brazil. In Western countries where genotype 3 (HEV‐3) predominates, pregnancy outcomes are generally favourable, contrasting with the ALF rates linked to genotype 1 (HEV‐1) in Asia and Africa. This clinical disparity is supported by ex vivo models showing that HEV‐1 replicates more efficiently and induces greater tissue damage at the maternal‐fetal interface than HEV‐3 (Gouilly et al. 2018).

Nevertheless, HEV remains a threat to other vulnerable groups in South America, as demonstrated by a report of HEV‐induced acute‐on‐chronic liver failure (ACLF) in an Argentine patient with underlying alcoholic liver disease (Fantilli et al. 2021).

HEV is a non‐enveloped, single‐stranded RNA virus belonging to the Hepeviridae family, genus *Orthohepevirus* A (Purdy et al. 2022). Of the eight known genotypes, HEV genotypes 1–4 are the primary cause of humans infectious. Genotypes 1 and 2 are prevalent in low‐income regions and transmitted via the faecal‐oral route, predominantly through contaminated water (Wang and Meng 2021). In contrast, genotypes 3 and 4 are more common in developed regions and primary transmitted through consumption of undercooked meat (especially from pigs and wild boars) or via direct contact with infected animals (Spahr et al. 2018). Other transmission routes include vertical (mother‐to‐child) transmission and parenteral exposure, such as through blood transfusion, organ transplantation, tattooing, or invasive medical procedures (Kamar et al. 2017; Geng et al. 2023).

Epidemiological studies have reported significant heterogeneity in HEV seroprevalence across populations. Identified risk factors included demographic characteristics (e.g., advanced 50 years, male sex), occupational or domestic exposure to animals (e.g., farmers, slaughterhouse workers, veterinarians) or socioenvironmental factors such as rural residence, lower educational level (Li et al. 2020; Songtanin et al. 2023).

In Brazil, the only HEV genotype isolated in humans, animals and environmental samples is genotype 3, which swine acting as the primary reservoir (dos Santos et al. 2011; Gardinali et al. 2012; de Oliveira‐Filho et al. 2017; Moraes et al. 2021). A systematic review estimated anti‐HEV IgG seroprevalence of 3% in the Brazilian general population (Tengan et al. 2019). Despite these findings, seroprevalence data remain limited and are primarily derived from specific subpopulations such as blood donors, rural or riverside communities and high‐risk groups (e.g., pregnant women and immunosuppressed patients) (Souza et al. 2019; Tengan et al. 2019; Cunha et al. 2022). A nationwide, household‐based serosurvey representative of the general population has not been conducted, representing a critical gap in the understanding of HEV epidemiology in Brazil.

Population‐based serological surveys are important tools for accurately determining the immune landscape of pathogens, understanding their transmission dynamics, evaluating the impact of vaccination programs, and guiding public health interventions (Metcalf et al. 2016). Between 2018 and 2019, we conducted a population‐based serosurvey in Recife, a large urban centre in Northeastern Brazil, to estimate the seroprevalence of arboviruses (Dengue, Zika and Chikungunya) (Braga et al. 2023). The stored samples and the associated detailed sociodemographic and household data from this survey provided a unique and efficient opportunity to investigate the seroepidemiology of HEV in an urban Brazilian population. Therefore, the objectives of this study were to estimate the seroprevalence of HEV and analyse risk factors associated with past infection among a general population of a metropolis in Northeastern Brazil.

## 2 Methods

### 2.1 Study Design and Location

A population‐based, descriptive cross‐sectional study was conducted in Recife, the capital city of the state of Pernambuco, in Northeastern Brazil.

Recife has an estimated population of 1,645,727 inhabitants and a territorial area of 218.843 km^2^, divided into 94 neighbourhoods distributed across six political‐administrative regions (Instituto Brasileiro de Geografia e Estatística 2024). It is located in the third most densely populated metropolitan area in the country and is considered the Brazilian capital with the highest level of social inequality, according to the last three national demographic censuses. Although spatial segregation of social classes exists in some areas, poverty and wealth coexist in close proximity within the same urban space (Instituto Brasileiro de Geografia e Estatística 2010). Approximately 40% of households in the municipality have a monthly per capita income of up to half the minimum wage, equivalent to about 136.4 US dollars, characterising a situation of social vulnerability affecting a substantial proportion of the population (Instituto Brasileiro de Geografia e Estatística 2023).

The reference population for this study consisted of residents of Recife aged 5 to 65 years who participated in a population‐based arbovirus survey conducted between 2018 and 2019 (Braga et al. 2023). Participants were selected through a two‐stage random sampling process within each neighbourhood. In the first stage, census tracts were randomly selected within each socioeconomic stratum based on the proportion of household heads with an income below two minimum wages, including those with no income, using data from the 2010 Demographic Census. In the second stage, households within the selected census tracts were sampled, and all residents within the eligible age range were invited to participate.

The 94 neighbourhoods of the municipality were classified into four homogeneous strata according to living conditions using a K‐means clustering algorithm, ensuring intra‐stratum homogeneity and inter‐stratum heterogeneity. Due to the small population size of the first and second clusters, which comprised neighbourhoods with higher household income, these were merged into a single high socioeconomic stratum. The third and fourth clusters were classified as intermediate and low socioeconomic strata, respectively.

The number of households to be selected in each census tract was determined based on an estimated average of 3.5 eligible residents per household. Within the randomly selected census tracts in each socioeconomic stratum, households were chosen using the Amostra Brasil package in R (R Core Team 2023). This tool performs simple random sampling of households and provides their geographic coordinates (Cordeiro et al. 2016). Sampling weights were calculated as the inverse probability of selection, adjusted for non‐response and applied in all analyses to ensure representative population estimates.

### 2.2 Data Collection

Data collection was carried out after participants were informed of the study's objectives and written consent term. Individual and household‐level data were obtained through interviews using a standardised questionnaire. While primarily designed for arbovirus surveillance, the questionnaire captured key environmental and socioeconomic indicators, such as housing characteristics, sanitary infrastructure and household income, which are relevant proxies for HEV zoonotic risk factors in urban settings. Subsequently, blood samples were collected and transported to the Virology and Experimental Therapy Laboratory at the Aggeu Magalhães Institute (IAM), Oswaldo Cruz Foundation (Fiocruz), where they were processed and stored at −80°C.

### 2.3 Laboratory Procedures

Anti‐HEV IgG antibodies were detected using a commercial ELISA kit (Euroimmun), based on recombinant antigens from hepatitis E virus genotypes 1 and 3, according to the manufacturer's protocol. Results were interpreted semi‐quantitatively by calculating the ratio of the sample's extinction value to that of calibrator 3. Samples with a ratio ≥ 1.1 were interpreted positive. All IgG‐positive samples were also tested for anti‐HEV IgM using the same manufacturer's ELISA kit (Euroimmun).

The Euroimmun assays were selected based on their previously demonstrated analytical and diagnostic performance (Pas et al. 2013; Vollmer et al. 2016). Comparative evaluations have shown that the anti‐HEV IgG Euroimmun assay exhibits high analytical sensitivity, with detection limits of approximately 1.5 IU/mL when calibrated against the WHO reference standard, and high diagnostic sensitivity during seroconversion. In addition, this assay provides quantitative IgG results traceable to the WHO standard, allowing improved comparability across studies (Vollmer et al. 2016).

### 2.4 Exposure Variables

Socioeconomic stratum—high, intermediate and low.

Individual characteristics—age in years (categorised: 5–24, 25–34, 35–44, 45–54, 55–65), sex (male/male), self‐reported skin colour (white, black, mixed race, other) and education level (elementary, high school and higher education).

Household characteristics—Dwelling type (apartment, house), sanitation system (public network, other, including septic tank, rudimentary cesspit, ditches, rivers, lakes), water supply (public network and others, including artesian well and water truck), waste disposal (public cleaning service or others, including dumpster, buried and burned) and head of household monthly income in minimum wage (~548 USD) (≤ 2, > 2 and < 4 and > 4).

### 2.5 Data Analysis

Data entry and management was performed through the REDCap electronic platform hosted at the University of Heidelberg, Germany.

We estimated the overall anti‐HEV seroprevalence and socioeconomic strata‐specific, with their respective 95% confidence intervals (95% CI). A descriptive analysis was conducted to examine the frequency distribution of individual and household characteristics.

The associations between exposure variables and HEV seropositivity were analysed using hierarchical multiple regression approach. First, univariable analysis was performed for each block of variables (individual and household characteristics) to calculate crude odds ratios (ORs) with 95% CIs. Variables associated with the outcome at a significance level of *p* < 0.20 in the univariable analysis were included in a multivariable logistic regression model for their respective block. Variables that maintained a statistically significant association within each block were included in a final combined multivariable model. Variable selection in all regression models was guided by Akaike Information Criterion (AIC) and *p*‐value. Goodness‐of‐fit for the final logistic regression model, which fitted data from a complex sampling design, was assessed using the Archer‐Lemeshow test. All estimates were weighted to account for the complex sample design, using the ‘survey’ package of the R program, version 4.1–1 (Lumley 2025).

The spatial distribution of anti‐HEV IgG positive cases was mapped using the SIRGAS2000 geodetic reference system (SIRGAS: The Geocentric Reference System for the Americas 2025). All statistical analyses were performed using Stata, version 15 and R software v. 4.0.2.

### 2.6 Ethical Issues

The study protocol was approved by the Research Ethics Committee of the Aggeu Magalhães Institute (IAM/Fiocruz Pernambuco; CAEE: 79605717.9.0000.5190; Approval No. 2.734.481) and the Hospital das Clinicas da Universidade Federal de Pernambuco (HC/UFPE; CAAE: 51880021.0.0000.8807; Approval No. 5.523.845). The study complied with Brazilian ethical guidelines (Resolution No. 466/2012 of the National Health Council). Written informed consent. For minors (ages 5–17 years), written assent was obtained in addition to the written informed consent provided by their legal guardians.

## 3 Results

Serological analysis was performed on samples from all 2,070 participants in the original study, of whom 416 (20.1%) were from the high, 726 (35.1%) from the intermediate stratum and 928 (44.8%) from deprived socioeconomic strata. Participants were predominantly female (58.5%), most self‐identified as mixed race (53.7%) and the majority had completed at least a high school education (62.0%) (Table 1). The largest age group was 5–24 years (30.0%), followed by 55–65 years (17.6%).

**TABLE 1:** Crude odds ratio (OR) of the association of the individual and household characteristics with HEV exposure. Recife, Northeast Brazil.

Characteristics	Total (*n* = 2070)	Positive	Negative	OR (95% CI)	*p*
		*n* (%)	*n* (%)		
*Individual*					
Age group					
05–24	621 (30.0)	6 (1.0)	615 (99.0)	1.00	**—**
25–34	323 (15.6)	16 (4.9)	307 (95.1)	4.94 (1.90–12.87)	**0.002**
35–44	375 (18.1)	16 (4.1)	359 (95.9)	4.14 (1.72–10.00)	**0.002**
45–54	387 (18.7)	15 (4.0)	372 (96.0)	4.00 (1.56–10.30)	**0.005**
55–65	364 (17.6)	21 (5.8)	343 (94.2)	5.85 (2.50–13.71)	**< 0.001**
Sex					
Female	1212 (58.5)	48 (4.0)	1164 (96.0)	1.00	—
Male	858 (41.5)	26 (3.0)	833 (97.0)	0.75 (0.46–1.23)	0.252
Skin colour					
White	592 (28.6)	22 (3.7)	571 (96.3)	1.00	—
Mixed	1113 (53.7)	35 (3.2)	1078 (96.8)	0.86 (0.53–1.40)	0.548
Black	320 (15.5)	16 (4.9)	304 (95.1)	1.35 (0.75–2.42)	0.320
Other	45 (2.2)	1 (2.0)	44 (98.0)	0.53 (0.07–3.98)	0.542
Education level^a^					
Fundamental	728 (35.2)	20 (2.7)	708 (97.3)	1.00	—
High school	776 (37.5)	34 (4.4)	742 (95.6)	1.65 (0.95–2.80)	0.079
University	507 (24.5)	18 (3.5)	489 (96.5)	1.27 (0.67–2.41)	0.472
*Household*					
Type of dwelling^b^					
Flat	413 (19.9)	17 (4.0)	396 (96.0)	1.00	—
Single‐store house	1655 (80.1)	57 (3.5)	1598 (96.5)	0.85 (0.51–1.43)	0.549
Sewage destination^c^					
Public network	1105 (54.1)	32 (2.9)	1073 (97.1)	1.00	—
Other destinations	937 (45.9)	41 (4.3)	896 (95.7)	1.49 (1.02–2.20)	**0.045**
Water supply^d^					
Public network	1780 (86.1)	60 (3.4)	1720 (96.6)	1.00	—
Other sources	287 (13.9)	14 (4.7)	273 (95.3)	1.40 (0.75–2.64)	0.296
Waste disposal^e^					
Household collection	1916 (92.6)	68 (3.5)	1848 (96.5)	1.00	—
Other	152 (7.4)	6 (4.1)	146 (95.9)	1.17 (0.57–2.41)	0.670

*Note:* Bold values represent the best statistically significant.

Abbreviation: 95% CI, 95% confidence interval.

^a^ 59 missing records.

^b^ 2 missing records.

^c^ 28 missing records.

^d^ 3 missing records.

^e^ 4 missing records.

Regarding housing characteristics, 80.1% single‐store homes, while 45.9% lacked connection to public sewage systems, relying instead on alternative sanitation methods (e.g., septic tanks, cesspits) (Table 1).

Of the 2.070 samples tested, 74 were positive for anti‐HEV (IgG), yielding an overall seroprevalence of 3.6% (95% CI: 2.9–4.3). None of the anti‐HEV IgG‐positive samples tested positive for IgM. Anti‐HEV IgG seroprevalence stratified by socioeconomic stratum was 3.3% (95% CI: 1.7–5.0) in the high stratum, 3.8% (95% CI: 2.6–4.6) in the intermediate stratum and 3.5% (95% CI: 2.4–4.6) in the deprived stratum.

**FIGURE 1:**
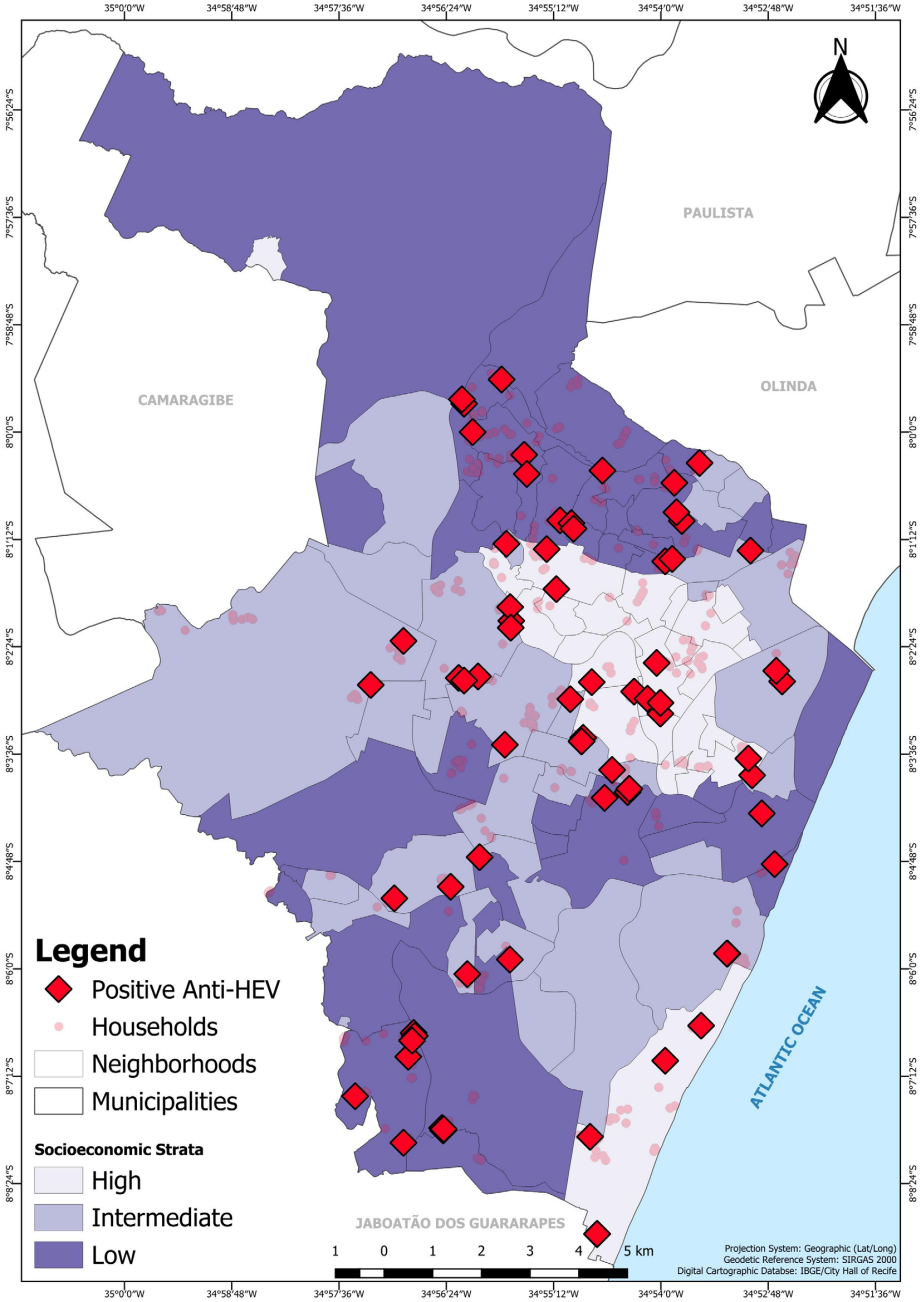
Spatial distribution of selected households and anti‐HEV‐positive residents in the municipality of Recife, Northeast Brazil.

Figure 1 shows the spatial distribution of HEV‐positive residents throughout the Recife municipality, showing that infections were widespread across all socioeconomic strata.

The results of the logistic regression analysis are presented in Table 1, crude associations and Table 2, final adjusted model. Table 2 presents all parameters of the final multivariable model, and its goodness‐of‐fit was confirmed by the Archer‐Lemeshow test (*p* = 0.559). In the univariate analysis, age group and sewage destination were significantly associated with HEV seropositivity (*p* < 0.05) (Table 1). In the adjusted final model (Table 2), age remained independent and strongly associated with HEV exposure. A clear dose–response association was observed, with the odds of exposure increasing with each successive age group. Adults aged 55–65 years had significantly higher odds of previous HEV exposure (adjusted OR = 6.02; 95% CI: 2.59–13.99; *p* < 0.001) compared to the reference age group (5–24 years). Furthermore, the type of sewage destination was independently associated with HEV positivity. Residents of households using alternative sewage systems (e.g., septic tanks, cesspits) had higher odds of HEV positivity compared to those connected to the public sewage network (OR = 1.58; 95% CI: 1.08–2.31; *p* = 0.021). No statistically significant associations were found between HEV exposure and sex, race/skin colour, education level, dwelling type, water supply or waste disposal in the adjusted model (all *p* > 0.05).

**TABLE 2:** Adjusted odds ratio (aOR) from final logistic regression model of the individual and household characteristics with HEV exposure including model fit parameters. Recife, Northeast Brazil.

Final model	Adjusted OR (95% CI)	*p*
Age group		
05–24	1.00	—
25–34	5.17 (1.95–13.73)	**0.001**
35–44	4.16 (1.72–10.02)	**0.002**
45–54	4.02 (1.55–10.42)	**0.005**
55–65	6.02 (2.59–13.99)	**< 0.001**
Sewage destination		
Public network	1.00	—
Other destinations	1.58 (1.08–2.31)	**0.021**

*Note:* Model fit statistics: Pseudo‐*R* ^2^ = 1.27; AIC = 622.67; Archer‐Lemeshow test *F* = 0.85 (*p* = 0.559); AUC = 0.665. Bold values represent the best statistically significant..

## 4 Discussion

This study, the largest population‐based study conducted in Brazil and the first survey in a major Northeast metropolis, Recife, found an overall HEV seroprevalence of around 3.6%. This percentage is close to the estimate for the general Brazilian population, about 3.0%, as determined by a systematic review with meta‐analysis (Tengan et al. 2019). Significant findings demonstrated a strong, dose‐dependent association with advancing age, with the highest levels of exposure observed in individuals aged 55 to 65 years, and a widespread distribution of HEV seropositive cases across all socioeconomic strata, city‐wide environmental risks.

However, information regarding the occurrence of HEV infection in Brazil remains scarce. This scarcity may be partly attributed to the limited availability of anti‐HEV testing within the Unified Health System (SUS), particularly at the primary healthcare level. In practice, access to anti‐HEV serological and molecular testing is largely restricted to reference laboratories operating within specific institutional and referral pathways, including laboratories affiliated with Fiocruz Ciência e Saúde pela vida (2025).

In Brazil, the first population‐based study to investigate the prevalence of anti‐HEV IgG in the population occurred in the city of São Paulo in 1998, through active search in randomly selected households from all districts and social strata of the municipality. It found an anti‐HEV prevalence of 1.68% among 1,059 individuals (Focaccia et al. 1998). Following this survey, two other population‐based studies were published. One in Rio de Janeiro, with an anti‐HEV prevalence of 2.4% among 699 residents of the Manguinhos community (Santos et al. 2002), and the other in a small municipality in São Paulo state, with a prevalence of 20% among 248 individuals (de Almeida E Araújo et al. 2020). This higher prevalence (20%) was attributed to potential environmental and zoonotic transmission factors, possibly linked to the invasion of wild boars in this small municipality in São Paulo that interbred with domestic pigs, facilitating viral spreading (de Almeida E Araújo et al. 2020).

Globally, according to a meta‐analysis, the anti‐HEV IgG seroprevalence ranged between 21.76% on the African continent, 7.28% in South America and 5.99% in Oceania (Li et al. 2020). In Latin America, the estimated anti‐HEV prevalence in the general population via a meta‐analysis was 9%, varying between 17% in Bolivia and 7% in Argentina and Brazil (Magri et al. 2025). A recent scoping review confirmed significant heterogeneity nationwide, with rates from 0.5% in the North to 59.4% in the South, the latter linked to cultural practices and pork consumption (Mariz et al. 2025).

In our study, multivariate analysis demonstrated that advanced age was independently associated with higher odds of prior HEV exposure. Adults aged 55 to 65 years exhibited significantly greater odds of previous exposure (aOR = 6.17) compared with the youngest age group (5–24 years). This apparent dose–response relationship reflects the cumulative risk of infection over time, a hallmark of endemic pathogen circulation, in which increasing age represents prolonged opportunity for exposure rather than an intrinsic biological susceptibility (Szklo & Nieto, 2019). Our findings corroborate seroepidemiological patterns observed both internationally (Takeda et al. 2010; Mansuy et al. 2011; Hartl et al. 2016; Li et al. 2020) and in Brazil (Passos‐Castilho and Granato 2017; Bricks et al. 2019; Zorzetto et al. 2021).

Distinct from the findings from some studies conducted in Brazilian populations (Souza et al. 2019; da Silva‐Sampaio et al. 2025), HEV seroprevalence did not differ by sex in our study population. In those reports, sex‐based differences have been attributed to behavioural risk factors, primarily occupational exposures such as swine farming, butchering, or slaughterhouse work and to factors like illicit drug use (Pereira et al. 2018; Costa et al. 2021; da Silva‐Sampaio et al. 2025). The lack of this association in our population suggests these specific high‐risk exposures were not primary drivers of transmission in our setting. This finding aligns with broader evidence from national and international meta‐analyses, which found no consistent sex‐based association for HEV. This indicates that any male bias is likely context‐specific rather than biological (Tengan et al. 2019; Li et al. 2020).

In contrast to patterns in which HEV infection is associated with markers of lower socioeconomic status (Li et al. 2020), we did not observe variation in seroprevalence across socioeconomic strata in our study. Consistently, a systematic review and meta‐analysis including 68 studies conducted in countries of the Americas reported that higher socioeconomic status did not confer protection against HEV infection (Horvatits et al. 2018).

The uniform spatial distribution of anti‐HEV seropositive cases across Recife suggests a city‐wide exposure risk rather than the presence of localised high‐risk areas. This homogeneous pattern, combined with the absence of a socioeconomic gradient, suggests that faecal–oral transmission may not be the primary route in this setting. Instead, the observed epidemiological profile is suggestive of a common source of exposure, such as zoonotic pathways, a pattern observed in other regions where HEV‐3 is endemic (Li et al. 2020). The seroprevalence of 3.6% identified in our investigation is comparable to rates reported in settings where foodborne zoonotic transmission is a major pathway (Kamar et al. 2017). While our study did not directly assess dietary habits, the consumption of meat and derived products from infected swine, whether from domestic farms or inadequately inspected slaughterhouses, remains a plausible hypothesis for HEV circulation in urban Brazil, as previously discussed in literature (Pereira et al. 2018).

This hypothesis is supported by both local and national evidence from the Northeast and Southern regions of Brazil (Heldt et al. 2016; de Oliveira‐Filho et al. 2017). Although no environmental surveillance for HEV has been conducted in Recife and large‐scale commercial pig farming is absent within the city's urban perimeter, the state of Pernambuco harbours a substantial swine reservoir. A study conducted in this state reported exceptionally high anti‐HEV IgG seroprevalence, reaching 81.2% (78/96) among slaughterhouse swine and 82.1% (188/229) across 16 farms (de Oliveira‐Filho et al. 2017), demonstrating a robust viral reservoir within the regional food production chain. Additional support for this interpretation comes from a study conducted in Southern Brazil, in which 36% of pork product samples tested positive for HEV (Heldt et al. 2016). Taken together, these findings indicate that the commercial food supply, sourced from infected swine in neighbouring regions, represents the most consistent explanation for the widespread human exposure observed in Recife.

Unexpectedly, residence in households using alternative sewage systems (e.g., septic tanks or rudimentary pits) was associated with a 60% increase in the odds of HEV seropositivity compared with households connected to the public sewer network. This finding contrasts with a study conducted among people living with HIV in Southern Brazil, which reported a negative association with similar sanitation conditions (aPR = 0.40) (Moss da Silva et al. 2019), underscoring the context‐specific nature of this relationship. In our urban setting, alternative sanitation systems likely do not represent a direct causal pathway but may act as proxies for broader infrastructural deficiencies. Such deficiencies may correlate with unmeasured risk factors for zoonotic transmission, including distinct food handling practices, increased contact with animals or contaminated environments, or reliance on unregulated food markets. This interpretation is supported by a survey among rural settlers in central Brazil, which reported a similar seroprevalence (3.4%) and suggested that unfavourable environmental condition, such as untreated water and inadequate sanitation, may increase HEV exposure over time (Freitas et al. 2017).

This study has some limitations that should be considered when interpreting the findings. The analysis was conducted on biobanked samples and data originally collected for a serosurvey designed to estimate arbovirus seroprevalence, which is typically higher than that of HEV. Consequently, while the sample size (*N* = 2,070) was sufficient to estimate HEV seroprevalence with precision, it provided limited statistical power for robust subgroup analyses and for detecting weaker associations in the multivariable model. However, it is important to note that the primary aim of the regression model was inference (to estimate measures of association) and not prediction. Therefore, while reported for completeness, predictive metrics like the AUC are of secondary relevance. The core limitation remains the precision of our effect estimates, reflected in the width of the confidence intervals. To overcome the specific limitation of potential model misspecification with complex survey data, we applied the Archer‐Lemeshow goodness‐of‐fit test (*F* = 0.85, *p* = 0.559), which confirmed the model was adequately calibrated. Nevertheless, the fundamental power constraints mean that non‐significant findings should be interpreted with caution rather than as definitive evidence of no effect. Future studies with larger sample sizes, preferably designed specifically for HEV, are needed to confirm these associations and explore other potential risk factors with greater power.

Another limitation was the lack of data on specific HEV risk factors. The original questionnaire was not designed to investigate zoonotic hepatitis and lacks information on critical exposures such as dietary habits (e.g., consumption of pork or game meat, cooking practices), direct contact with animals (especially swine), or occupational exposures. Furthermore, the cross‐sectional design precludes any assessment of causality or determination of the timing of infection.

Despite these limitations, the study has several methodological strengths that enhance the robustness and relevance of its findings for understanding HEV epidemiology in urban Brazil. The population‐based sampling design ensured representativeness and generalizability. In addition, the multi‐stage cluster sampling strategy, stratified by socioeconomic level, was specifically designed to capture the pronounced social inequalities characteristic of the study setting. The high participation rate and the application of sampling weights to account for the complex survey design further strengthen both the internal and external validity of the results.

In conclusion, this serological survey demonstrated the circulation of HEV in the general population of a metropolitan area in Northeastern Brazil, with higher seroprevalence observed among older individuals and those residing in households not connected to the public sewage system.

These findings offer valuable evidence to inform public health strategies in Brazil, particularly regarding the strengthening of surveillance and regulation of swine breeding, slaughtering practices and the commercialization of pork products. Nevertheless, additional studies conducted in metropolitan areas of other regions, especially in the North and South, as well as in smaller municipalities, are necessary to achieve a comprehensive epidemiological understanding of HEV infection in Brazil.

## Author Contributions

*Conceptualization*: Carolline A. Mariz, Cynthia Braga, Wayner V. Souza, Maria de Fatima P. Militão de Albuquerque, Edmundo Pessoa Lopes. Data curation: Carolline A. Mariz, Cynthia Braga and Edmundo Pessoa Lopes. *Formal analysis*: Carolline A. Mariz, Cynthia Braga, Wayner V. Souza, Carlos F. Luna and André Luiz Sá de Oliveira. *Funding acquisition*: Cynthia Braga, Wayner V. Souza, Maria de Fatima P. Militão de Albuquerque and Clarice N.L. de Morais. *Investigation*: Carolline A. Mariz, Cynthia Braga, Elisa de Almeida Neves de Azevedo and Edmundo Pessoa Lopes. *Methodology*: Carolline A. Mariz, Cynthia Braga, Wayner V. Souza, Maria de Fatima P. Militão de Albuquerque, Clarice N.L. de Morais and Edmundo Pessoa Lopes. *Project administration*: Carolline A. Mariz and Cynthia Braga. *Supervision*: Carolline A. Mariz, Clarice N. L. Morais and Cynthia Braga. *Validation*: Carolline A. Mariz, Cynthia Braga, Wayner V. Souza, Maria de Fatima P. Militão de Albuquerque, Clarice N.L. de Morais and Edmundo Pessoa Lopes. *Writing – original draft*: Carolline A. Mariz, Cynthia Braga, Wayner V. Souza, Maria de Fatima P. Militão de Albuquerque, Clarice N.L. de Morais and Edmundo Pessoa Lopes. *Writing – review and editing*: Carolline A. Mariz, Cynthia Braga, Wayner V. Souza, Carlos F. Luna, André Luiz Sá de Oliveira, Elisa de Almeida Neves de Azevedo, Clarice N.L. de Morais, Maria de Fatima P. M Albuquerque and Edmundo Pessoa Lopes.

## Funding

This work was supported by the National Council for Scientific and Technological Development (Conselho Nacional de Desenvolvimento Científico e Tecnológico—CNPq; grant numbers 304413/2022‐4 to CB and 302696/2021‐0 to MFPMA; Notice FACEPE PPSUS/SES/PE—06/2020, no.: APQ‐0396‐2.11/20 to CNLM).

## Ethics Statement

This study was approved by the Research Ethics Committee of the Aggeu Magalhães Institute, Pernambuco, Brazil (CAEE no. 79605717.9.0000.5190; approval number: 2.734.481) and the Research Ethics Committee of the Hospital das Clinicas da Universidade Federal de Pernambuco (HC/UFPE; CAAE: 51880021.0.0000.8807; Approval No. 5.523.845). Research data were collected after participants (or their legal guardians if under 18) were informed about the study's objectives and provided written informed consent, in accordance with Brazilian National Health Council Resolution No. 466/12. Participants aged 5–18 also provided oral and/or written assent.

## Conflicts of Interest

The authors declare no conflicts of interest.

## Supporting information


**Data S1:** Seroprevalence and risk factors for hepatitis E virus in a metropolis of northeastern Brazil: a population‐based survey.

## Data Availability

All relevant data are within the manuscript and its Supporting Information S1.
