# Supplementary material for: Seroprevalence and Risk Factors for Hepatitis E Virus in a Metropolis of Northeastern Brazil: A Population‐Based Survey
Source: Zoonoses Public Health. 2026 Mar 9;73(4):326–35. doi: 10.1111/zph.70050 (PMC13144426; doi:10.1111/zph.70050)
Supplement: Supplementary file 1 — Data S1: Seroprevalence and risk factors for hepatitis E virus in a metropolis of northeastern Brazil: a population‐based survey. [file ZPH-73-326-s001.docx]

**Seroprevalence and Risk Factors for Hepatitis E Virus in a Metropolis of Northeastern Brazil: A Population-Based Survey**

Carolline A Mariz, Cynthia Braga, Wayner V Souza, Carlos F Luna, André Luiz Sá de Oliveira, Elisa de Almeida Neves de Azevedo, Clarice N L de Morais, Maria de Fatima P M Albuquerque, Edmundo Pessoa Lopes.

**standardized questionnaire**

| Individual  characteristics |  |  |
| --- | --- | --- |
| [indi_date] | Date of interview | text (date_dmy, Min: 2018-08-01, Max: 2019-02-28), Required  Field Annotation: @HIDEBUTTON |
| [indi_id] | Individual Number | text (number) |
| [lab_sample_reg] | Lab sample registry | text (number) |
| [age] | What is your age? | text (number, Min: 5, Max: 93), Required |
| [dob] | What is your date of birth? | text (date_dmy, Min: 1900-01-01, Max: 2018-12-31) Field Annotation: @HIDEBUTTON |
| [sex] | Sex | 1 Male  0 Female |
| [race] | What is your color/race? | 1 White  2 Black  3 Asiatic  4 Mixed  5 Indigenous  8 Do not know/ Not informed |
| [education] | What is your highest education degree? | 1 1° year of pre-primary education  2 2° to 5° year of primary education, incomplete  3 2° to 5° year of primary education  4 6° to 9° year of lower secondary education, incomplete  5 Lower secondary education, complete  6 Upper secondary education, incomplete  7 Upper secondary education, complete  8 Academic degree, incomplete  9 Academic degree, complete  10 Ignored (Do not know)  11 Not applicable (< 6 years old or has never gone to school) |
| [household_head] | Head for the family (household) | 1 Yes  0 No |
| [income] | What was your income last month? | 1 ≤ 2 mw (minimum wage)  2 2-4 mw  3 4-10 mw  4 10-20 mw  5 > 20mw  8 Not Known/Not informed  9 No income |
| Household characteristics |  |  |
| [household_id] | Household ID | text |
| [cluster] | Cluster | 1 Cluster 1  2 Cluster 2  3 Cluster 3 |
| [census_tract] | Census_tract | text (number, Min: 000001, Max: 399999), Required |
| [resident_count] | How many residents live in this household? | text (integer, Min: 1, Max: 100) |
| [residents_5_65] | How many residents aged between 5 and 64 years? | text (integer, Min: 1, Max: 100) |
| [type_house] | Type of House | 1 Brick house  2 House in a condominium  3 Apartment  4 Tenement  5 Tent  999 Other  8 Not Known/ Not informed |
| [type_house_other] Show the field ONLY if: [type_house] = '999' | Specify: | text |
| [bathroom_waste] | The bathroom waste is discarded in | 1 General or pluvial sewer  2 Septic fosses  3 Rudimentary fosses  4 Ditch  5 River, lake or sea  999 Other  8 Not Known/Not informed |
| [bathroom_waste_other] Show the field ONLY if: [bathroom_waste] = '999' | Specify: | text |
| [water_supply] | What is the water supply used in the house? | 1 General water distribution  2 Water well  3 Water well outside the property  4 Water tank truck  5 Rainwater stored in cistern  6 Rainwater stored in another way  999 Other  8 Not Known/Not informed |
| [water_supply_other] Show the field ONLY if: [water_supply] = '999' | Specify: | text |
| [garbage] | How is the garbage discarded in this house? | 1 Removed and collected by public cleaning system  2 Temporarily placed in a public cleaning system container  3 Burned  4 Buried  5 Discarded in a wasteland / sidewalk  6 Discarded in a river, lake or sea  999 Other  8 Not Known/Not informed |
| [garbage_other] Show the field ONLY if: [garbage] = '999' | Specify: | text |
